# 

**Published:** 1876-12

**Authors:** 


### INDEX.

### A.

Abscesses, carbolic acid in opening 306
Abnormal fœtation.............. 396
Abortion, retained placenta in.... 292
Absorption power of milk....... 507
Acids, vegetable................ 176
Acute diseases, digitalis in ...... 481
Acute articular rheumatism...... 553
Acidulated gargles in typhoid fever 89
Adams, Dr. W. A., valedictory address.................... 142
Advertising, remarks on..188, 437, 510
Address on relation of medical editors to the profession........ 449
Adhesive plaster, a new.......... 508
Aged, sedatives for.............. 624
Albumen, to detect in urine...... 697
Alum, tannin and oxide of zinc in sticks... .................. 699
Albuminaria, management of..... 600
Albuminaria, chronic, iodide of potash in.................. 46
Albuminaria in pregnancy (scarlatinal, 51).................... 239
Albuminaria, galic acid in....... 295
Albuminaria, treatment of....... 375
Alcohol, for medicinal use........ 68
Alcohol, in glandular swellings... 103
Alcohol, poisoning by............ 505
Alcoholic phthisis............... 108
Alcoholism, treated by strychnine 369
Alkalies, effect upon the blood.... 110
Alkaline injections in chronic diseases of stomach............. 741
Alley, A. K., M.D., on firwein (opium poisoning, 268)...... 394
Ammonia, in rheumatism......... 237
Ammoniated tinc. guiac in sore throat...............613, 433, 708
Ammorrhœa, simple.............. 726
American Medical Association.443, 245
Amyl nitrite in nervous diseases.. 545
Anal vegetations.... ......... ... 169
Anderson, L. B., M.D., on sanguinaria canadensis............. 11
Anæmia, pernicious............. 173
Anæmia, cerebral............296, 542
A new medicine................. 180
A new medicine, "Seven Spring's Mass"....................11, 705
Antispasmotic cough mixture..... 380
Anti-rheumatic mixture.......... 432
Antiseptic dressing, new......... 498
Antidote, multiple............... 685
Antiseptic, chloral as an.... .... 570
Anesthesia, surgical, in children.. 433
Anesthesia by mesmerism........ 697
Anodyne, local.................. 435
Anurism, involving the innomenata, etc.................... 503
Animal vaccination.............. 569
Angina pectoris, treatment of.... 735
Apocyinum cannabinum in dropsies......................... 81
Apomorphia, hypodermically, as an emetic...............171, 369
Aphonia........................ 378
Arsenic, rules for administering.. 427
Arsenic, in children..... ...... 92
Aromatic sulphuric acid, in necrosis ................... ...... 601
Asthma, uterine................. 102
Asthma, belladonna in........... 691
Asthma, recipe for.............. 739
Aspiration, in intestinal obstruction 417
Atropia, in phthisical sweating... 223
Atropia sulphate, in meningitis... 394
Atropia sulphate as an antidote to prussic acid................. 558
Atropia in cystitis..... ......... 620
Atropia, substitute for.. ......... 752
Aural discharges, salacytic acid in 212
A word for the South—Med. Rec. 117

### B.

Batte, G. H., M.D., on ammorrhœa and dysmenorrhœa.......... 226
Baths of chloral in small pox.... 241
Baths, in summer complaints..... 163
Baths, tepid, in fevers........... 176
Bathing in intero-colitis.......... 619
Bandaging, in mammary abscess. 496
Ballard, the French Prof...... ... 436
Barry, W. A., M.D., on placenta prævia..................... 579
Barr, W. F., M.D, old and new remedies................ ...... 386
Barr, W. F., M.D., on Seven Springs Mass.... ................ 705

Baxter, G. A., M.D., on pelvic examinations.................. 147
Bell, A. N., M.D., address before Editor's Association......... 449
Belladonna and opiates............ 242
Belladonna, in asthma.............. 691
Bed sores, to prevent..........430, 508
Beef, extract of................... 699
Blackurn, J. C., M.D., on type of diseases, etc................ 7
Blackberry brandy, or cordial... 379
Bleeding, in acute diseases....... 342
Bladder to inject without a catheter................ .... 654
Bloodless operation, duration of.. 78
Bloodless operation, new method. 751
Booth, John W., M.D., on post-paitem hemorrhage.......... 712
Boracic acid in ring worm........ 105
Borax, solubility of in glycerin... 86
Boils and carbuncles, mercurial ointment in................. 176
Boracic acid, for wounds......... 500
Brain, physiology of............. 239
Bran, box, in fractures.... ...... 358
Bran bread question......... .... 690
Breathing, the lungs............. 227
Breath, offensive.......... ...302, 311
Bright's disease..............176, 551
Bromide of potassium in convulsions....................... 230
Bromide of potassium, local use of........ ..............466, 671
Bromide of potassium, in milk as a vehicle.................... 698
Bromide of lithium..........240, 307
Bromide of camphor........... ... 308
Bromide of ammonia,in hemiplegia 183
Bromide of iron and its preparations.... ...................... 494
Bromide of iron, pills of......... 494
Bromide of iron, syrup of...... ... 494
Bromine, solution of, as a dressing 296
Bromohydrate of quinine........ 86
Bronchitis, digitalis in........... 94
Bronchial catarrh................ 731
Bronchus, calculus of............ 490
Bronchocele, treatment by alcohol 566
Burns, extensive, treatment of.232, 310
Burns, dressing for...........312, 509
Burns, new remedy for.......... 426
Burns, lime glycerine for......... 111
Burns, local treatment of......... 627
Buflalo lithia waters.... ....... 190
Burnet, Swan M., M.D., medical observations in Europe...... 513

### C.

Calomel in small doses, in syphilitic headache... ............ 560
Calculus of the bronchus......... 490
Camphor water, formula for..... 183
Camphorated tincture of opium.. 426
Camphor and water, in constipation.. ....................... 229
Camphor, in erysipelas.......112, 436
Camphorated ether, in erysipelas.......... ............. 508, 699
Cancer, eucalyptus globulus in... 345
Cancer, Dr. Wm. Parker on...... 477
Cancer, offensive discharges from.. 380
Cancer, citric acid in.........184, 581
Capillary puncture, in tympanitis. 747
Carbuncle... .......... ....166, 176
Carbuncle, strapping for.... . 111
Carbuncle, carbolic acid in....... 626
Carbuncle, recipe for..... ....... 749
Carbolic acid in diabetes........ 67
Carbolic acid in rheumatism..... 371
Carbolic acid and hypophosphites, in scarlatina................ 94
Carbolic acid, dangerons from absorption......... ......:.418, 493
Carbolic acid in whooping cough. 691
Carbolic acid,as a local anesthetic.. 306
Carbolic acid, therepeutics of..... 614
Carbolic acid, in piles............ 659
Carbolic acid, in carbuncle.... .. 626
Carcinoma....................... 538
Carious teeth, collodion in....... 81
Castor oil........................ 571
Case of constitutional hemorrhage 755
Cathartic pill, improved......... 379
Catarrh snuff.................... 509
Cathetreism, in lasceration of the urethra..................... 303
Catarrh remedy, Sage's.......... 565
Catarrh, nasal, treatment of...635, 687
Catarrh, bronchial............... 731
Caustic, precaution in using...... 696
Caustic, in diseases of the cervix.. 306
Cautery, galvanic................ 283
New....... ...... ..... 425
Cauterizing the throat, caution in. 434
Cement....... ................ 435
In wood and iron.......... 436
Centennial.... ................. 439
Cervix, dilatation of in dysmenorrhœ................. ...... 179
Cerebro-spinal meningitis, heat in 462
Cerebro-spinal fever............ 173
Cerebral anæmia................ 542
Chafing......................... 432
Chase, Dr. S. D., on cups, etc., in gastritis....... ............ 657

Chemical discoveries............ 243
Chenery, Dr. E, on diphtheria... 521
Chicken pepsin.................. 579
Chinoidine, pills of, for chills.... 80
Chills and fever, chloroform in... 96
Chloride of soda, in cancer...... 397
Chlorine water, in the bite of rabid animals.................. 310
Chloral........................ 428
Chloral, suppositories of......172, 301
Chloral, enemata of.............. 419
Chloral, in strychnine poisoning.. 70
Chloral, in ptyriasis............. 374
Chloral for ulcers...............71, 784
Chloral hydrate, in pruritus vulvæ.......................361, 369
Chloral, in nocturnal emesis...... 184
Chloral in ozena................. 570
Chloral as an antiseptic.......... 570
Chloral in hydrophobia........... 744
Chloral, fatal result from injection of.......................... 694
Chloral in vomiting of pregnancy. 696
Chloral and iodide of potash, incompatable.................. 424
Chlorate of potash............... 427
Chlorate of potash, in diarrhœa 306, 632
Chlorate of potash, in gastro-intestinal catarrh.................. 506
Chlorosis, liquor potassæ in...... 675
Chloroform, in malaria.......... 96
Chloroform and morphine ...... 69
Chloroform, in infants...111, 305, 633
Chloroform, in dyspepsia......... 428
Chloroform, in urticaria.......... 562
Chloroform, as a preservative ag't. 309
Chloroform, mode of administering 678
Chloroform, in hydrocele......... 508
Chorea, arsenic in............... 92
Chorea, phosphate of zinc in..... 92
Chorea, treatment of............. 743
Cholera infantum, prophylactic in......................90, 422
Cholera infantum.688, 650, 679, 501, 585
Cholera, epidemic, Bibliography of 121
Choleate of soda, to prevent gall stones.................... 566
Chronic gastro intestinal catarrh.. 506
Chronic folicular pharangitis..... 151
Chronic ulcers of the leg......... 175
Chronic pneumonia of the apex, in children.................. 763
Cicuta, in scarlet fever.......... 535
Cinquo-quinine, strychnia and iron 426
Citric acid, in cancer........184, 581
Cipripedium, in ammorrhœa..... 726
Citrate of quinoidine............. 632
Cleft palate, treated without operation....................... 415
Clysters, in diphtheria........... 565
Coal oil......................... 691
Coca, action of.................. 572
Cod liver oil, emulsion of........ 484
Cod liver oil, mixture............ 484
Cod liver oil with iron........... 485
Cod liver oil and lacto-phos. iron....................181, 485
Cod liver oil ...............507, 510
Cod liver oil injections........... 569
Cold, extreme effects of.......... 428
Cold pack, Dr. Hurt on.......... 60
Cold, use of in fever............. 425
Cold, in scarlatina............... 75
Cold water hypodermically to relieve pain..................... 508
Cold water in typhoid fever...85, 98
Cold in the head................ 569
Cold water, in summer complaint. 571
Collodion, in carious teeth....... 81
Compound liquorice powder...... 499
Complementary parts of disease... 641
Concentrated vegetable medicines. 278
Constipation, chronic............ 177
Constipation, treatment of.259, 229, 617
Constipation and diarrhœa, water and camph., in...........383, 415
Convulsions of children, veratrum in......................... 337
Convulsions arrested by posture...................503, 553
Convulsions, bromide of potash in. 239
Consultations.................... 383
Conium......................... 634
Contagionists, a nut for.......... 572
Corns, cure for.................. 504
Coryza, treatment of............. 64
Coston, John, M. D., cure of abnormal fœtation................ 396
Cosmetic powders, harmless...... 504
Cotton wool, use of in coryza and sore throat.................. 90
Coto bark, in diarrhœa, etc....... 569
Cough mixture, anti-spasmodic... 380
Cough of phthysis, formula for... 376
Cracks in stoves, to close......... 381
Crayons of tannin............... 501
Creasote in hemorrhages....... .. 491
Creasote in diarrhœa............ 433
Creasote to administer 106, in tape worm..................... 107
Croton oil paint in cellulitis...... 752
Croton chloral.................. 89
Cups and ice in gastritis.......... 657
Curious incompatability.......... 424
Cyanides in rheumatism......... 567
Crystaline lens, discharged by a blow....................... 193

### D.

Datura, as a substitute for atropia.................... 571, 752
Depaul, Prof., of Paris, remarks on 362
Decimal system................. 436
Delirium tremens................. 242
Dentrifices.......................... 509
Dentition at seventy-three....... 434
Diarrhœa, vaso-parylitic......... 306
Diarrhœa, perses-quinetrate of iron in........................ 399
Diarrhœa, hemorrhagic, salacin in 410
Diarrhœa, creasote in............ 433
Diarrhœa, camphor and water in. 229
Diarrhœa of typhoid fever, triat.. 473
Diarrhœa, in children............ 486
Diarrhœa, imperfect mastication as a cause....................... 504
Diarrhœa of infants, chlorate of potash in.................... 632
Diabetes, opium in.............. 372
Diabetes, novel treatment of..... 96
Diabetes, milk diet in............ 104
Diabetes, sulph. calcium in...... 566
Diabetes, glycerine in............ 693
Diabetes, carbolic acid in........ 67
Diascorea, tincture of........... 278
Diagnosis of fissures of the anus in infants........................ 545
Diagnosis of heart affections..... 82
Diagnostic sylabus of uterine diseases, etc.................... 582
Dietetics........................... 209
Digitalis, in pneumonia.......... 410
Digitalis, in bronchitis........... 94
Digitalis, in typhoid fever....... 294
Digitalis, in acute diseases....... 481
Digitalis.......................... 305
Diphtheria. 112, 235, 363, 368, 378, 380 407, 625, 6g5, 683
Diphtheria successfully treated. 521, 540
Diphtheria, tracheotomy in....... 491
Diphtheria, hyposulphite soda in. 552
Diphtheria, liquor potassæ in.... 554
Diphtheria, clysters in........... 565
Diphtheria, salacylic mixture in.. 572
Diphtheria, anti-septic treatment of 622
Diphtheria, muriate of ammonia in 624
Diphtheria, formula for.......... 636
Diphtheria, treat. of .......... . 742
Diphtheria, and filth ........... 205
Diphtheria, treated with sulphate of iron.......................... 754
Diphtheritic sore throat........ .. 756
Diphtheritic paralysis............ 673
Diseases in Middle Georgia, type of 7
Diseased joints, sulphuric acid in. 72
Diseases, complementary parts of. 641
Disinfecting powders, universal... 698
Doctors of Pharmacy............ 319
Doctors, the making of............ 435
Dowel, Dr., his treat. of hernia... 703
Drunkenness, cure for............ 416
Dropsy of the amnion........... 29
Dropsies, eucalyptus in........... 597
Dropsies, apocynum cannabinum in.......................... 81
Duncan, Dr. D. P., on quinine in parturition..................... 729
Dysentery...................10, 45, 508
Dysentery, salacylic acid in...... 300
Dysentery, ipecac in.........170, 348
Dysentery, abortive treat. of. ... 423
Dysentery, chronic...........157, 747
Dysentery, topical treatment of... 550
Dysentery, formula for........... 636
Dyspepsia, chloroform in......... 428
Dyspepsia, cases of............... 707
Dysmenorrhœa, hysterical—recipe for.......................432, 433
Dysmenorrhœa, dilatation of cervix in........................ 179
Dysmenorrhœa, cepripedin in.... 726
Dysmenorrhœa and sterility...... 732

### E.

Ear, removal of foreign bodies from................184, 368, 687
Earth closet..................... 189
Easley, Dr. E. T., in fever....... 721
Eczema, lime water in........... 506
Eczema, in children............. 242
Eclampsia, treatment of.......... 622
Editorial communications........ 113
Electricity, in skin diseases....... 304
Electrolysis in nævi.............. 492
Electrolytic treatment of tumors.. 536
Electricity, in hyperpyrexia. .... 91
Electricity, its therapeutical uses.. 538
Electro-therepeutics.............. 17
Electricity, in jaundice........... 175
Emetic for children, apomorphia.. 364
Emerson, Dr. E. W., in cholera infantum......................... 650
Enuresis................ 429, 502, 737
Enemata of chloral, new method. 419
English, pure...................... 507
Entero-coletis, bathing in........ 619
Enteric fever, sulphurous acid in.. 685
Epidermis, formed by transplanting hair.................... 749
Epilepsy.......................... 545
Epilepsy, trephining in........... 185
Epistaxis...............378, 561, 569
Epistaxis, plugging for........... 295
Ergot, as an anti-pyretic......174, 308

Ergot, in intestinal hemorrhage... 309
Ergot, new preparation of ..... 375
Ergot, in gallactorrhœa and mastitis........................ 490
Ergot, internally for epistaxis.... 561
Ergot, for hæmoptysis............ 572
Erasive soap.................... 507
Erysipelas, nitrate of lead in..... 740
Erysipelas, silicate of potash in... 377
Erysipelas, camphorated ether in............... .......508, 699
Erysipelas, camphor in........112, 436
Erysipelas, veratrum in....... .... 280
Erythema nodosum. .......... ... 687
Eserine, in tic. ...... .............. 96
Esmarch's bandage for chronic ulcers.................... ... 561
Esmarch's bandage, new application........................ 751
Esmarch's method............... 266
Ether spray, in chorea............ 743
Ethical puzzle.................. 373
Eucalyptus globulus, in cancer... 345
Eucalyptus globulus, medical value of.. . .... ....... 379
Eucalyptus, in dropsies.......... 597
Evans, Rev. C. A., address....... 129
Evils against the profession...... 114
Expectorant mixture............ 746
Experiments .................. 436
Extract of malt.................. 189
Extract of beef................. 699
Eyes, hydrastis in affections of.... 280

F.

Felon......................... ..... 166
Feet, sweating................... 366
Feet perspiration, to prevent..... 744
Feet, fetid........ .. ......... 751
Fevers, tepid baths in.......... 176
Fever, physiological antagonism.. 721
Fever, cold in.................. 725
Firwein ........................ 394
Fistulo in ano, new operation for.. 95
Fishures of the anus, diagnosis.. 545
Flesh, shower of in Kentucky.... 409
Foot powder .................. 507
Freckle lotion................... 184
Fractures, improved method for.. 304
Fractures, treated by the bran box 358
Fracture, treatment of......... . 756

G.

Gall bladder, tapping the......... 502
Galvanic cautery............ ....... 283
Galic acid, in scarlatinal albuminaria........................ 295
Gallactorrhœa and mastitis, ergot in......................... 490
Gall stones, choleate of soda in... 566
Ganglion of the wrist........... 106
Gangrene, treated with salacylic acid.... .................... 376
Gangrene, idiopathic ........... 269
Gargle for sore throat............. 613
Gargle, quinia as a........... .. 370
Gastric vertigo.... . .......... 543
Gastritis, acute, cups and ice in... 657
Gastralgin, nitrite of amyl in..... 65
Gelseminum.................... 552
Gelseminum in poison oak eruption 741
Georgia Medical Association..... 313
Glandular enlargements, hyperdermic treat . ..................... 684
Glandular swellings, alcohol in... 103
Glue, liquid..... ............... 429
Glycerine, in diabetes......... ... 693
Glycerole of sabacetate of lead.634, 695
Gout, cure of.................... 687
Gout, diet for..................... 682
Goitre.......................... 537
Gonorrhœa, kava in............. 686
Gonorrhœa, sandle wood in...... 693
Gonorrhœa, hydrastin in......... 78
Gonorrhœa, mixture for.......... 757
Gonorrhœal rheumatism... ..... 420
Gonorrhœa, as a cause of sterility. 204
Gonorrhœa, treatment of ...... 238
Gonorrhœa, quinine injection for.. 737
Gossypium herbaceum ... ...... 267
Granulations, exhuberant, carbolic acid in..................... 615
Grindelia robusta ........171, 307 429
Gum arabic mucilage............ 505
Guiacum in throat diseases..... .. 496
Gunshot wounds................ 15
Gynæcology, advances in 1875.... 631

H.

Hæmoptises, poulticing in....... 568
Ergot in ............... ..... 572
Hæmaturia and quinine.......... 728
Hair restorative..... ........371, 503
Hair falling off, to prevent.... ... 431
Hair tonic..................... 93
Handy, Dr. D. S., on gunshot wounds ..................... 15
Hanks, Dr. J. A.. letter from..... 186
Hernia, new treatment of........ 267
Headache of the decline of life.... 406
Headache.......................88, 110
Headache, nervous............... 96
Headache, sick, remedy for...... 508

Headache, syphilitic............ 560
Heat, in cerebro spinal meningetis 462
Heat, use of in menorrhagia...... 82
Heart, examination of........... 58
Heart affections, diagnosis of..... 87
Helonias dioica................... 529
Hemorrhage, intestinal, treatment of. ........................ 310
Hemorrhage, creasote in......... 491
Hemorrhage, uterine............ 234
Hemorrhage, post partem........ 712
Hemorrhage, perchloride of iron in 717
Hemiplegia, strychnine in........ 488
Hemiplegia, bromide of ammonia in ........................ 183
Hemicrania......... .......... 545
Heredity, illustration of......... 470
Hip joint, test line in injury of... 293
Hip joint disease, treatment of.... 301
Histology and histrochemistry of man.......... .. ........... 637
Hæmatoxylum, in alkalinity of urine.................. ..... 360
Hodge, Dr. G. D., on hæmaturia and quinine.................. 728
Homicidal handwriting.......... 562
Hooping cough ...... ......632, 681
Hot water douche, in neuritis..... 495
Hot packing, in rheumatism...... 424
Hurst, Dr. W. R., singular worm case........................ 50
Hydrangia, concentrated tinct. of. 279
Hydrastin, in gonorrhœa........ 78
Hydrastis, tincture of....... .... 280
Hydrate of chloral, in labor...... 48
Hydrate of chloral in strychnia poisoning................... 70
Hydrate of chloral, external use of 667
Hydrate of chloral, for ulcers.... 71
Hydrate of chloral to prevent chill 673
Hydro chloral, by the rectum in vomiting .. ....... ....... 696
Hydrocele, chloroform in........ 568
Hydrophobia, specific for.....488, 546
Hydrophobia, injection of chloral for ...................... 744
Hydrophobia, xanthium spinosum in.. ..................... 750
Hyasciamin, for vomiting in pregnancy...................... 750
Hypertrophy of prostrate, treatment of................ .... 489
Hyperpynexia, electricity in...... 91
Hyposulphite of soda, in diphtheria 552
Hypodermic injections, in enlarged glands............ ... ..... 684
Hypochondriasis, sexual......... 178
Hysteria and dysmenorrhœa...... 433
Hysterical attacks, to arrest...... 79

### I.

Ice bags in pericarditis.......... 294
Ice medicated, application of..... 366
Ice, medicated, in scarlatina...... 233
Imposed upon.................. 245
Incompatability of iodide and chlorate of potash... ...... 688
Incontinence of urine .......429, 568
Indellible marking paper......... 190
Infants, chloroform in........ ... 111
Infantile vomiting............... 380
Injecting bladder, new method of. 654
Intermittent fever, steam bath in.. 66
Intestinal atony.................. 550
Intestinal obstruction ...... 230, 417
Inunction. ...................... 109
Iodide potassium with carb. ammonia. ..................... 95
Iodide pottassium to conceal the taste......................... 95
Iodoform........ .............. 417
Iodoform with iron as an alterative 605
Iodoform, as a topical application 609
Iodoform pencils............ ... 431
Iodoform, odourless............. 539
Iodoform, preparation of......... 564
Iodoform in vaginismus.......... 234
Ipecac in dysentery............170, 348
Iritis, diagnosis of............... 225
Iritis, grandelia robusta in........ 429
Itch, recipe for.................. 79
Itch, treatment of..... ......... 184
Itinerant pile surgeons, their new plan....................... 659

### J.

Jaborandi....... ...........373, 560
Jaborandi in Cholera and Scarlatina......................... 408
Jaborandi, Sialagogue, effect of... 551
Jaborandi, Sialagogue, effect to prevent.................. ... 570
Jalap Biscuit.................... 509
Jaundice, Electricity in.......... 175
Jervia, a new Alkaloid........... 312
Joints, neurosis of............... 483

### K.

Kava-kava in gonorrhœa......... 686
Kenan, Dr. Thomas H., case of occlusion vaginæ............ 194
Kenan, Dr. Thomas H., on trephining an epileptic.......... 195
Kilpatrick, Dr. A. K., cases in practice..................... 196

Kilpatrick, Dr. A. K., Texas Medical Association.............. 257
Kind words........................ 117

L.

Labor, difficult, new expedient in. 109
Lead, colic in Paris hospitals..... 236
Lead poisoning, new source of.... 565
Leather........................ 243
Legislating, vs. quack medicines.. 436
Leeches, to make bite............. 633
Leg, ulcers of.................175, 555
Leptandra as a purgative......... 420
Letter from a co-laborer......119, 251
Letter from Dr. Hanks........... 186
Leucocythæmia.................. 183
Leucorrhœa, hydrastis in......... 280
Leucorrhœa, permanganate potash in...................... 612
Lewis, Dr. J. M., vesicants in children.................... 334
Lemon tonic.................... 745
Lithium, bromide of............. 756
Lime water in eczema .......... 506
Lime glycerine, for burns........ 111
Lime juice, and pepsin........... 374
Lipscomb, Dr. W. L., on sterility from gonorrhœa.............. 204
Liquid glue.................... 429
Liquor opii sedativous........... 492
Liquor potassæ, in diphtheria.... 554
Liquors, taste, recipe to remove.... 564
Liquor potassæa, in chlorosis..... 675
Liquor potassæa, in obesity...... 364
Liver medicine, Simmons'....... 700
Local anodyne.................. 435
Local treatment of skin diseases.. 593
Longevity of physicians......... 365
Loomis' Tonic.................. 699
Lungs, breathing. .............. 227
Lupuline, in delerium tremens.... 242
Lyon, Dr. A. A., on opium habit. 200

M.

Malarial fevers, to prevent........ 265
Malarial hæmaturia.............. 264
Mammæ, puerperal diseases of. 154, 165
Mammitis and mammary abcess, bandaging for............... 496
Man, comparative physiology of.. 243
Mania-a-potu................... 495
Manual of diet on health, review of 638
Marshall Hall's nerve tonic...... 433
Masturbation, in children........ 54
Masturbation, to prevent......... 682
McBride, Dr. Alexander, on diseases, etc.... ............... 641
Medical Congress, International.. 270
Medical Societies, remarks on.... 315
Medical education............... 317
Medical Association of Alabama--1875—'76.................... 319
Medical College of Georgia, address........................ 129
Medical editors, relation of to the profession. ................. 449
Medical observations in Europe... 573
Membranes, rupture of........... 303
Menses, in phthysical patients.... 681
Menstruation, painful........506, 570
Menstruation, irregular cases of... 708
Meniers disease................ 568
Mesmitic annesthesia............. 697
Metric system in prescriptions.... 381
Metrorrhagia, heat in............ 87
Milk, Nestle's Mothers........... 435
Milk absorption, power of........ 507
Milk diet in diabetes............. 104
Milk sickness.................. 350
Milk as a vehicle for bromide of potash........................ 698
Milk supply, stoppage of......... 748
Milk, secretion of, to prevent...156, 610
Milk vender punished............ 634
Miscarriage, hypodermic use of morphia in.. ...........289, 751
Mistura glycyrrhiz a, com........ 499
Mistura ferri chloride, com....... 242
Mistura asthmatica.............. 739
Mistura Bumstead .............. 751
Multiple antidote............... 685
Morphia, hypodermically in miscarriage...................... 289
Mucilage.............. ......435, 505
Muriate ammonia in diphtheria.... 624
Muriate ammonia in enlarged spleen........................ 696
Myringitis, attropia in .......... 364
Myelitis, a sequel of diarrhœa.... 491

N.

Nail, surgical.................. 107
Nail, biting, to cure.............. 362
Nævi, electrolysis in............ 492
Nævus, or port wine marks, to remove........................ 603
Nasal catarrh...........219, 411, 687
Nasal polypus, chloride of iron in. 175
Nasal cavities, plugging in........ 295
Nausea from opiates, belladonna in...................... ... 242
Necrosis, aromatic sulphuric acid in ......................... 601

Nerve tonic.................... 433
Nervous and mental disease, amyl in.......................... 545
Nervous deafness, strychnine in... 94
Neuralgia, formula for........... 695
Neuralgia, phosphide of zinc in... 91
Neuralgic affections, nitrite of amyl in.......................... 95
Neuralgic affections, eseraine in.. 96
Neuralgia obstinate, pills for..297, 375
Neuritis, hot water in........... 495
Neutral sulphate of atropia, hypodermically................. 241
Neuroses of the joints.......... 483
Nipples, sore, and ulcerated..112, 308, 505, 630
Nipples, ulcerated and fissured.. 743
Nipples, sore, picric acid for..... 733
Nitrate of zinc, pencils of........ 373
Nitrate of silver stains, to remove 505
Nitrate of lead, in erysipelas.... 740
Nitirte of amyl.................. 734
Nitrite of amyl in gastralgia..... 65
Nitrite of amyl in neuralgia...... 95
Nitrite of amyl in hiccough...... 80
Nitric acid, in uterine practice.... 297
Nocturnal emesis, chloral in...... 184
Nurses sore mouth............... 77

O.

Obesity, sea water in........... 689
Obesity, liquor potassæ in........ 364
Offensive discharge from cancer, etc 380
Offensive perspiration............ 500
Oil of butter.................... 578
Oil of eggs in sore nipples and chaps......................... 505
Oil of red cedar, poisoning by... 749
Oil of sandalwood in gornorrhœa. 693
Ointment, resolvent...........502, 509
Oleum ovorum................... 499
Old friends with new faces...... 161
Old and new remedies........... 386
Onion as a diuretic.............. 631
Opium in pregnancy and parturition........................... 1
Opium habit..................... 200
Opium poison, cure of........... 269
Opium in diabetes.............. 372
Opium, Battey's sedative, solution of.......................... 492
Opethalamia, purulent.......... 281
Orchitis, treatment of........... 300
Osuteri, rigidity of.............. 526
Os uteri, excoriation............ 618
Ottorrhœa, styptic cotton in...... 81
Ottorrhœa, salacine in........... 353
Ovariotomy, no more............ 497
Ozone, powder to produce... 571, 699

P.

Pain, relieved by cold water hypodermically................ 508
Palpitations of the heart, to arrest 174
Pancreatic meat, emulsion....... 636
Pate, Thin, M. D., dots by the way side....................... 577
Paralysis diphtheritic............ 673
Parametritis and perimetritis.... 311
Paraphimosis.................... 581
Parturition, quinine in.......... 729
Pelvic examinations, per rectum.. 147
Pelvic cellulites, croton oil paint in 752
Pepsin, chicken................. 579
Permanganate of potash, in leucorrhœa......................... 612
Pericarditis..................... 294
Peruvian bark, in sore throat..... 168
Pernicious anæmia.............. 173
Perchloride of iron, in post partem hemorrhage................... 717
Perspiration, offensive.......... 500
Perityphlitis, when to operate for. 746
Persesquinitrate of iron, in diarrhœa......................... 399
Pharmacy in Atlanta Medical College........................ 437
Phagedinic sores................ 240
Philadelphia, editors visit to..... 439
Phosphide of zinc............... 91
Phosphide of zinc in neuralgia and chorea......................... 91
Phosphide of zinc in spinal irritation.......................... 91
Photography as a means of diagnosis....................... 566
Phthysical sweating, atropia in... 223
Phthysis, alcoholic.............. 108
Phthysical patients, neuroses in.. 681
Phymosis, modified operation for. 288
Physicians, longevity of......... 365
Physicians' call book and tablet. 123
Physiological antagonism in treat, 721
Phytolacca, tincture of.......... 729
Picric acid for sore nipples....... 721
Piles, diagnosis and treatment of.....................215, 659
Pill, improved cathartic......... 379
Pimples on the face............. 696
Pitting, in variola, to prevent.... 567
Pityriasis, chloral in............ 374
Placenta, retained............183, 292
Placenta previa... 579, 547, 321, 414
Plethysmograph, 549, digitalis in. 410
Pneumonia, in children......... 356
Podophylum, tincture of......... 279

Poisons, antidote for .. ...... .. 299
Poison oak, tincture of........... 379
Poisoning by santonin. ......... 421
Poisoning by alcohol............ 505
Poisoning by red cedar........ .. 749
Poison oak eruption.......... ... 741
Poisoning by lead, new source of. 565
Polyuria, treatment of........... 742
Post partem hemorrhage......... 712
Posture in convulsions ......... 552
Potassium iodide, incompatable with chloral...... ..... ... 688
Powell, Dr. T. S., on truths and facts...................... 246
Powder for producing ozone..... 699
Pregnancy, vomiting in......105, 487
Priapism...................... 687
Prolapsus recti, treatment of .... 734
Prophylactic in cholera infantum. 422
Prophylamin in rheumatism..... 101
Prostate enlarged, cathetre in.... 84
Prostate enlarged, mistaken for calculus.................... 93
Prostate, fibroid tumor of........ 628
Prostatic hypertrophy............ 489
Pruritus vulvæ.......85, 369, 558, 690
Pruritus, vinegar for............ 184
Pruritus vulvæ in pregnancy..228, 430, 502
Pruritus vulvæ, chloral hydrate in 361
Pruritus ani and vulvæ.......... 432
Pruritus hiemalis, treatment..... 557
Prussic acid, antidote for.... ... 558
Psoriasis, tar for............... 421
Psychology of man.... ........ 243
Puerperal fever, proehylactic treat. of......................... 556
Puerperal insanity.............. 39
Puerperal diseases of the mammæ. 165
Pumpkin seed in tape worm...... 377
Purpura, treatment of........... 532
Purpura hemorrhagica........... 174

Q.

Quack medicines, legislating against... ................. 436
Quinine, new use of............. 365
Quinine gargle in sore throat..... 370
Quinine, in surgical practice..... 548
Quinine, in Menier's disease...... 568
Quinine, in whooping cough..... 681
Quinine, in hæmaturia.......... 728
Quinine, in parturition.......... 729
Quinine, injection of, in gonorrhœa 737

R.

Raw meat, to preserve.......... 636
Raw meat, use of................ 86
Raw meat, to make palatable. ... 311
Reflex contraction, atropia in.... 241
Remedies, old and new.......... 386
Remittent fever, treatment of..... 677
Resolvent ointment.............. 502
Retrospective glances........... 766
Rheumatism, cyanides in ....... 567
Rheumatism, acute.............. 689
Rheumatism, ice in..........488, 533
Rheumatism, gonorrhœal........ 420
Rheumatism, hot pack in........ 424
Rheumatism, mixture for........ 432
Rheumatism, salacylic acid in.... 312
Rheumatism, treatment of.231, 335, 567
Rheumatism, carbolic acid in.... 371
Rheumatism, propylamin in...... 101
Rheumatism, a certain cure for... 298
Rheumatism, ammonia in.... ... 237
Rheumatism, xanthoxylum in.... 281
Rheumatic fever, cold bath in.... 100
Rhus taxicodendrum, antidote for 108
Rigidity of the os uteri.......... 526
Ringworm, borax in.... ....... 105
Rivers, Dr. G. M., on cancerous ulcers...................... 397
Rupturing the membranes....... 303

S.

Sage's Catarrh Remedy.......... 565
Salacin, in attorrhœa........ .... 353
Salacin, in hemorrhac diarrhœa.. 410
Salacylic acid.................. 500
Salacylic acid, in aural discharges 212
Salacylic acid, solution of....739, 752
Salacylic, in offensive breath..302, 311
Salacylic acid, in dysentery...... 300
Salacylie acid, in rheumatism.567, 312
Salacylic acid, syrup of.......... 380
Salacylic acid, in gangrene...436, 376
Salacylic acid, in diphtheria...50, 572
Salacylic acic as a local disinfectant...................... 62
Sanguinaria canadensis.......... 11
Santonine, poisoning by......... 421
Scabies, recipe for........... ... 79
Scarlatina.......... ...75, 94, 233, 665
Scarlatina, prophylactic in....... 64
Scarlatina, sulpho carbolate, soda in.....................363, 694
Scarlet fever, cicuta in.......... 535
Sciatica and constipation, remedy for......................... 695
Science and religion............ 244
Scientific gleanings............. 243
Sea water in obesity............ 689
Scrofula, tubercle from.......... 104
Scrofulous sores.............312, 315

Sebaceous tumors................ 538
Sedatives for the aged............ 624
Septic inoculations....... ....... 430
Seven Springs Mass......180, 705, 706
Sexual hypochonidriasis.......... 178
Sheet zinc, to prevent bed sores.. 430
Shoulder presentations, version in. 291
Silicate of potash in erysipelas... 377
Sims, Dr., and the ethics.......... 445
Sick headache, remedy for........ 508
Sialagogue effect of jaborandi.... 570
Simmons' liver med., formula of.. 700
Skimble-Scamble dots by the wayside......... ... .. ... 578
Skin diseases, local treatment of.. 593
Skin diseases, oil of maze in..... 570
Skin grafting.................... 267
Skin diseases, electricity in...... 304
Skin diseases, juniper fumigations in........................ 736
Sleeplessness, cure of.... ....... 708
Small pox, chloral hydrate in..... 241
Smith, T. Curtis, M.D., on placenta previa.................. 321
Smith, T. Curtis, M.D., on opium in pregnancy, etc............ 1
Smith, T. Curtis, M.D., on sulphide of lime................ 391
Snow as a fertilizer............. 244
Soap liniment. ................. 549
Social and scientific progress, a summary.................... 34
Sodic sulpho carbolate.......... 363
Soft hands....................... 368
Sore nipples........... .....488, 112
Sore throat, peruvian bark in....... 168
Special notice.................. 768
Spiritus frumente rectificatus..... 67
Spectroscope.................... 244
Spider web in intermittents...... 541
Spinal irritation, phos. zinc in... 92
Spleen enlarged, mur. ammon in. 696
Sprains, treatment of....418, 496, 676
Starch, iodid, antidote to poison.. 299
State Board Health .............. 320
Steam bath in intermittent fever.. 66
Stains of nitrate of silver, to remove...................... 505
Stains of clothing, erasive soap for 507
Sterility from gonorrhœa......... 204
Sterility from dysmonorrhœa..... 732
Stokes' expectorant mixture...... 746
Stomach, chronic disease of...... 741
Stomach, diseases of, treated with stomach pump............. 623
Stomatitis matena................ 77
Stomatitis, ulcerative, remedy for. 419
Stoughton's bitters.............. 430
Stove polish as an irritant........ 503
Stoves, cracks in to close.... ...
Strangury, cantharidal... .......
Stricture of urethra.............. 663
Strychnia subcutaneously for nervous deafness................ 94
Strychnia, as an antiemetic....... 112
Strychnia poisoning, chloral in... 70
Strychnia, in hemiplegia.......... 488
Strychnia vs. alcohol... ........ 369
Strychnia vs. opium..... ... ... 370
Styptic cotton, in ottorrhœa...... 81
Subcutaneous injection for tumors 168
Subcutaneous use of carbolic acid in rheumatism........ ..... 371
Subacatate of lead, glycerole of... 634
Sulphite of cadmium ...... .... 98
Sulphate of cinchonidia......688, 745
Sulphide of calcium..........391, 566
Sulphate of atropia, for reflex contraction................. 241
Sulpho carbolate of sodium.64, 530, 694
Sulpho carbolate of zinc in gonorrhœa....................... 616
Sulphuric acid in diseased joints.. 72
Sulphurous acid in enteric fever....................551, 685
Sulphuric acid............ .... 568
Summer complaint, cold water in......... ..... 163, 175, 571
Suppositories of chloral......172, 301
Suppression of urine....... .... 431
Suppurative discharges, quinine in 365
Surgical nail.... ....... ....... 107
Surgical instruments, patents on.. 511
Surgical anesthesia in children.... 434
Sweats, coliquitive, to check.... 740
Sweating feet, remedy for........ 366
Syphilitic headache, small doses calomel in................. 560
Syphilis, tinc. stillingia, a formula 278
Syphilitic buboes, treatment...... 735
Syrup of coffee..... ............ 95
Syrupus cinchonæ alcoholicus.... 689

### T.

Tannin, local use of........ ..... 57
Tannin, crayons of.............. 50
Tannin, dressing for wounds...... 623
Tapping the gall bladder......... 502
Tape worm, pumpkin seed in.... 377
Tape worm, rational treatment of. 416
Tape worm, pomegranite bark in.. 563
Tape worm, creasote in... ...... 107
Tape worm, case of.............. 738
Tape worm, remedy for..... .... 739
Tar in psoriasis.... ........... 421
Taste for liquors, recipe to cure... 564
Tedious labor, venisection in..... 196

Teething infants.................. 435
Testicle inflamed............... .. 431
Tetanus, traumatic............. .. 265
Tetanus, uterine.................. 227
Tetanus in the horse.............. 633
Texas Medical Association........ 257
The Sunny South.................. 770
Throat, inflammation of, remedy.....................496, 613
Tinea dicalvans ....... ........ 302
Tincture of poison oak........... 379
Tincture chloride of iron in nasal polypus .................. . 175
Tincture stillingia in syphilis..... 278
Tincture deascoria, concentrated. 278
Tonic, for intermittents.......... 740
Tonic, lemon...................... 745
Tonic, Loomis'.................... 699
Tonsils, enlarged, prophylactic in. 633
Tonsilitis..... ............... ... 436
Tonsilitis, bromide in............ 671
Toothache........................ 570
To our advertising agents........ 769
Tracheotomy in diphtheria....... 491
Transactions of Medical Association of Alabama for 1876..... 639
Trechinosis ..................... 266
Transactions Medical Society of West Virginia................ 123
Truss, elastic.................... 190
Truths and facts ................ 246
Trycophyton, treatment of........ 548
Tumors, subcutaneous treatment of 168
Tubercular matter in food, infections......................... 73
Tubercle from scrofula........... 104
Tumors subcutaneously treated... 536
Tumors sebaceous......... ...... 538
Tympanitis, capillary puncture for 747
Typhoid fever.................... 611
Typhoid fever, acidulated gargles in 89
Typhoid fever, cold water in..... 85
Typhoid fever, digitalis in........ 294
Typhoid fever, cause of........... 299
Typhoid diarrhœa, treatment of.. 473
Typhoid or enteric fever, sulphuric acid in................ .... 685

### U.

Ulcers, chronic, iodoform of iron in.......................... 605
Ulcers, chronic, formula for...... 286
Ulcers and wounds, dressing for.. 667
Ulcers, chloral for.... ....... ... 748
Ulcers, fistulous.................. 275
Ulcers at Charity Hospital........ 285
Ulcers, chronic, of the leg........ 555
Ulceration of frænum linguæ in whooping cough....... .... 748
Ulceration stomatitis, recipe for.. 419
Urethra, stricture of............... 662
Urethra, female, dilatation of..... 427
Urethra, lacerated cathetreism in. 303
Urethra, rupture of... ........... 272
Urethral blenorrhagia ............. 87
Urine, incontinence of........... 568
Urine, albumine in, to detect..... 697
Urine, alakalinity of, hœmatoxylum in.... ................. 360
Urine, suppression of............ 431
Urtecaria, treatment of........... 562
Uterine disorders......... ....... 700
Uterine elevator, Barrington's.... 190
Uterine hemorrhage............. 234
Uterine sound, when not to use... 370
Uterine tonic, nervine and alterative... ............... ...432, 433
Uterus, diseases of............... 287
Uterus, diseases of, caustic in..... 306
Uterus, diseases of, nitric acid in.. 297
Uterus and vagina, inflammation of.... ....................... 582

### V.

Vaccination, animal.............. 569
Vagina, occlusion of...... ...... 194
Vaginismus, iodoform in..... ... 234
Valedictory address, by Dr. Adams 142
Vanellin.......................... 367
Vanswieton's liquor.............. 698
Variola, to prevent pitting in..... 567
Varix............................ 87
Vascular, naevi, to cure.......... 603
Vasiline, what it is.............. 309
Vegetable acids................... 176
Vegetable medicines.... ........ 278
Venerial sores, gangrenous, treatment of......................... 240
Venisection in tedious labor...... 196
Veratrum in convulsions of children............... ........ 334
Veratrum, tincture of............ 280
Version in shoulder cases, contra indications.................. 291
Vertigo, gastric.................. 543
Vesicants, in diseases of children.. 334
Vick's Floral Guide............... 769
Vinegar in pruritus.............. 184
Vomiting in pregnancy..105, 112 487, 696
Vomiting, infantile............... 380
Vomiting, remedy for.... ...... 112

### W.

Warts, cure for................... 504

Water proof varnish for paper.... 434
Water, Dr. Curry on remedial use of........................ 59
Watson, J. C., M.D., on paraphimosis........................ 581
Whooping cough, carbolic acid in 692
Whooping cough, quinine to abort 681
Whooping cough, ulceration of frænum in................... 748
Wilson, Dr. D. C., on idiopathic gangrene.................... 269
Wood and iron to cement........ 436
Word, Dr. R. C., scientific gleanings........................ 243
Worm, discharged through abdominal wall................... 50
Writing and reading............. 425

X.

Xanthoxylum, tincture of, in rheumatism...................... 281
Xanthoxylum spinosum in hydrophobia...................547, 750

Y.

Yellow fever in Savannah........ 576

### Colaborators and Contributors.

GEORGIA.

T. S. Mitchell, M.D.
J. M. Shaw, M.D.
J. N. Sheny, M.D.
T. M. Tules. M.D.
G. D. Case, M.D.
W. H. Hall, M.D.
S. G. White, M.D.
J. C. Wisdom, M.D.
J. E. Pope, M.D.
E. H. W. Hunter M.D.
Thomas Burdell, M.D.
L. B. Bouchelle, M.D.
W. C. Musgrove, M.D.
W. L. M. Harriss, M.D.
J. M. Tullis, M.D.
G. S. Ullman, M.D.
John Coster, M.D.

KENTUCKY.

Charles Kearns, M.D.
W. S. Taylor, M.D.
Douglass Morton, M.D.

TEXAS.

L. R. Lincecum, M.D.
T. J. Heard, M.D.

TENNESSEE.

F. K. Bailey, M.D.
F. A. Ramsey, M.D.
J. D. Tucker, M.D.

WEST VIRGINIA.

I. . Berry, M.D.
N. D. Tobey, M.D.
P. H. Clark, M.D.
A. L. Knight, M.D.

IOWA.

C. H. Tidd, M.D.
harles H. Lathrop M.D.

ALABAMA.

J. J. Grace, M.D.
J. D. Rush, M.D.
A. C. Edwards, MD.
S. B. Hogan, M.D.
J. B. Barnett, M.D.
J. C. Knox, M.D.

NORTH CAROLINA.

R. S Hallsey, M.D.
John W. Booth, M.D.
E. A. Anderson, M.D.
George A. Foot M.D.
H. Kelly, M.D
A. B. Pierce, M.D.
S. S. Satchwell, M.D.
J. B. Hughs, M.D.
Patrick Booth, M.D.

SOUTH CAROLINA.

G. M. Rivers, M.D.
E. T. McSwain, M.D.

OHIO.

W. P. Wells, M.D.
J. C. Bishop, M.D.
E. H. Trickle, M.D.
W. W. Fierce, M.D.
J. B. Graves, M.D.
J. M. Lucas, M.D.

NEW YORK.

J. S. Baily, M.D.

Ely Van DeWalker, M.D.

Lewis Balch, M.D.

W. H. Vail, M.D.

OREGON.

W. W. Cusick, M.D.

NEW JERSEY.

P. A. Harriss, M.D.
Jno. D. McGill, M.D.
D. M. Forman, M.D.

VIRGINIA.

J. N. Upshur, M.D.
L. B. Anderson, M.D.
W. F. Barr, M.D.
E. A. Drewry, M.D.
S. P. Ripley, M.D
J. S. Wharton, M.D.
R. J. Preston, M.D.
E. Horner, Jr., M.D.

MISSISSIPPI.

E. Cutter, M.D.

T. H. Mayo, MD.

I. H. Payne, M.D.

J. M. Lewis, M.D.

A. G. Smythe, M.D.

W. T. Beall, M.D.

W. L. Lipscomb, M.D.

Robert Kel[illegible] D.

E. T. Henr[illegible].

T. P. Lockwood, M.D.

S. V. D. Hill, M.D.

Edward Lee, M.D.

ARKANSAS.

C. D. Hodge, M.D.
A. W. Hobson, M.D.
J. K. Hodge, M.D.
A. D. Hodge, M.D.

INDIANA.

A. Patton, M.D.
G. A. Dyer, M.D.
J. M. Williamson, M.D.

